# Supplementary material for: Daphnetin Alleviates Bleomycin-Induced Pulmonary Fibrosis through Inhibition of Epithelial-to-Mesenchymal Transition and IL-17A
Source: Cells. 2023 Dec 8;12(24):2795. doi: 10.3390/cells12242795 (PMC10742308; doi:10.3390/cells12242795)
Supplement: Supplementary file 1 [file cells-12-02795-s001.zip › cells-2720063-supplementary.pdf]

Table S1. Sequence of primers and size of genes

Genes	Sense (5' to 3')	Antisense (5' to 3')	Size (bp)
β-actin (mouse)	GGCTACAGCTTCACCACCACA	CGGATGTCAACGTCACACTT	280
IL-17A (mouse)	CAGCAGCGATCATCCCTCAAAG	CAGGACCAGGATCTCTTGCTG	301
IL-6 (mouse)	TTCCATCCAGTTGCCTTCTT	CAGAATTGCCATTGCACAAC	200
RORgt (mouse)	CGCACCAACCTCTTTTCACG	CAGCTCCACACCACCGTATT	276
Col1a1 (mouse)	GCAAGAGGCGAGAGAGGTTT	GACCACGGGCACCATCTTTA	270
αSMA (mouse)	TCCTGACTGAGCGTGGCTA	GTTTCGTGGATGCCCGCTG	255
GAPDH (human)	CTCTGCTCCTCCTGTTCGAC	TTCCCGTTCTCAGCCTTGAC	270
Col1α1 (human)	AGTGGTTTGGATGGTGCCAA	ACCCTGGGGACCTTCAGAG	294
αSMA (human)	TCCCTGAACACCACCCAGTG	GTGCTTCGTCACCCACGTA	251
